# Hepatitis B virus genotypes, expression quantitative trait loci for *ZNRD1-AS1* and their interactions in hepatocellular carcinoma

**DOI:** 10.18632/oncotarget.9854

**Published:** 2016-06-06

**Authors:** Zhenzhen Liu, Ci Song, Juan Wen, Lu Xu, Yao Liu, Jian Zhu, Liguo Zhu, Zhibin Hu, Hongxia Ma, Li Liu

**Affiliations:** ^1^ Digestive Endoscopy Center, The First Affiliated Hospital of Nanjing Medical University, Nanjing, China; ^2^ Department of Epidemiology and Biostatistics, School of Public Health, Nanjing Medical University, Nanjing, China; ^3^ Jiangsu Key Laboratory of Cancer Biomarkers, Prevention and Treatment, Collaborative Innovation Center of Cancer Medicine, Nanjing Medical University, Nanjing, China; ^4^ Nanjing Maternity and Child Health Care Institute, Nanjing Maternity and Child Health Care Hospital Affiliated with Nanjing Medical University, Nanjing, China; ^5^ Pathology Center and Department of Pathology, Soochow University, Suzhou, China; ^6^ Qidong Liver Cancer Institute, The First People's Hospital of Qidong, Qidong, China; ^7^ Department of Infection Diseases, Jiangsu Province Center for Disease Prevention and Control, Nanjing, China

**Keywords:** HCC, susceptibility, eQTL, HBV genotype, interaction

## Abstract

Genetic variants in zinc ribbon domain-containing 1 antisense RNA 1 (*ZNRD1-AS1*) have been reported to be associated with development of hepatocellular carcinoma (HCC). We sought to determine the influences of *ZNRD1-AS1* polymorphisms and their interactions with Hepatitis B virus (HBV) genotypes on the risk of HCC. In this study, we conducted a large population case-control study with 1,507 HBV-related HCC cases and 1,560 HBV persistent carriers. Three single-nucleotide polymorphisms (SNPs) in *ZNRD1-AS1* (rs3757328, rs6940552 and rs9261204) were genotyped using a TaqMan allelic discrimination assay, and the HBV genotypes were identified by multiplex PCR. We found consistently significant associations between the *ZNRD1-AS1* rs6940552 and rs9261204 SNPs with an increased risk of HCC (additive genetic model: adjusted OR = 1.16, 95% CI = 1.03-1.32 for rs6940552; adjusted OR =1.20, 95% CI = 1.06-1.35 for rs9261204) and found a borderline association between rs3757328 and HCC risk. Besides, we observed a dose-dependent relationship between increasing numbers of variant alleles of the SNPs and HCC risk (*P* for trend <0.001). Moreover, we observed a stronger combined effect of the three SNPs on HCC risk among the subjects infected with non-B genotype HBV (adjusted OR = 1.26, 95% CI = 1.05-1.50) compared with HBV B-related genotypes (adjusted OR = 0.89, 95% CI = 0.69-1.15; *P*= 0.029 for heterogeneity test). We also found that a multiplicative interaction between the variant alleles and the HBV genotype significantly affected HCC susceptibility (*P* = 0.030). Together, these results indicate that *ZNRD1-AS1* may influence HCC risk accompanied by HBV genotypes.

## INTRODUCTION

Hepatocellular carcinoma (HCC) poses a substantial threat to public health, particularly in less developed regions [1]. More than 50% of HCC cases occur in China [2]. It has been estimated that 75–85% of HCC cases in China are attributable to chronic HBV infection. However, only a minority of chronic HBV carriers eventually develop HCC [3], thus highlighting the importance of genetic susceptibility in HBV-related HCC.

Different genotypes of HBV are defined when the entire genome displays a sequence divergence of > 8%. HBV genotypes have distinct geographic distributions, and the incidence of HCC also varies in different regions of the world [4]. HBV genotypes have been found to differ in terms of clinical liver diseases and disease outcomes [5, 6]. However, because of small sample sizes, low success rates of HBV typing and different study designs, the previously reported effects of HBV genotypes on the outcomes of persistent HBV infections have varied greatly.

Zinc ribbon domain-containing 1 (ZNRD1) is a zinc finger-related protein cloned from human leukocyte antigen (HLA) [7]. HLA is an important control gene for immune characteristics, and previous data have demonstrated that HLA polymorphisms exhibit certain correlations with the outcome of HBV infections and the development of HCC [8, 9]. Additionally, ZNRD1 has been analyzed as a host cellular factor that influences virus replication [10]. Interestingly, as a transcription-associated gene, *ZNRD1* has recently been found to be a novel negative modifier in carcinogenesis [11, 12]. These findings highlight the importance of *ZNRD1* in the development of HBV-related HCC.

*ZNRD1* is located on chromosome 6p21.3. There is a long non-coding RNA (lncRNA) *ZNRD1-AS1* in the upstream region of *ZNRD1*. Using bioinformatic analyses, we have previously determined that three SNPs (rs3757328, rs6940552 and rs9261204) in *ZNRD1-AS1* may be expression quantitative trait loci (eQTLs) for *ZNRD1* (http://www.regulomedb.org) [13, 14]. The significant associations of the three *ZNRD1* eQTLs SNPs in *ZNRD1-AS1* with the risks of both chronic HBV infection and HCC have been tested in our previous study. Our previous *in vitro* experiments have also demonstrated that *ZNRD1* knockdown inhibits the expression of HBV mRNA and promotes the proliferation of HepG2.2.15 cells [15]. Hence, we hypothesized that *ZNRD1* eQTL SNPs may influence the HCC risks associated with HBV genotypes. To test this hypothesis, we performed a case-control study to evaluate the effects of the HBV genotype, *ZNRD1* eQTL SNPs and their interactions on HCC risk.

## RESULTS

The demographic characteristics of the 1,507 HBV-related HCC patients and the 1,560 persistent HBV carriers have been summarized previously [16]. There were no significant differences in the distributions of age or gender between the HCC patients and the persistent HBV carriers (*P* = 0.835 and 0.687, respectively). The HBV genotypes B, C, BC (coinfection), and D were identified in the subjects of this study through nested multiplex PCR and sequencing, as previously described [16].

The genotype distributions of the SNPs rs3757328, rs6940552 and rs9261204 in the HCC cases and the persistent HBV carriers are described in Table 1. The observed genotype frequencies of the three SNPs in the HBV persistent carriers were all in Hardy-Weinberg equilibrium (*P* = 0.721 for rs3757328, *P* = 0.723 for rs6940552 and *P* = 0.971 for rs9261204). The logistic regression analysis with an additive genetic model indicated that the variant alleles rs6940552 and rs9261204 increased the host's HCC risk compared with the persistent HBV carriers (adjusted OR = 1.16, 95% CI = 1.03-1.32 for rs6940552; adjusted OR =1.20, 95% CI = 1.06-1.35 for rs9261204). Moreover, a borderline significant association was observed between rs3757328 and the HCC risk (adjusted OR = 1.11, 95% CI = 0.96-1.28; Table 1).

**Table 1:** Associations between three SNPs and HBV-related HCC susceptibility

Genotype	HCC patients	HBV persistent carriers	OR (95% CI)	*P*
	(n = 1507)	(n = 1560)		
	N (%)	N (%)		
rs3757328				
G/G	1038 (71.9)	1146 (73.8)	1.00	
G/A	362 (25.1)	375 (24.1)	1.07 (0.90-1.26)	
A/A	43 (3.0)	33 (2.1)	1.44 (0.91-2.29)	
Dominant			1.10 (0.93-1.29)	0.262
Recessive			1.42 (0.90-2.25)	0.134
Additive			1.11 (0.96-1.28)	0.150
rs6940552				
G/G	958 (64.2)	1048 (67.5)	1.00	
G/A	461 (30.9)	453 (29.2)	1.11 (0.95-1.30)	
A/A	73 (4.9)	52 (3.4)	1.54 (1.07-2.22)	
Dominant			1.16 (1.00-1.35)	0.055
Recessive			1.49 (1.03-2.14)	0.031
Additive			1.16 (1.03-1.32)	0.018
rs9261204				
A/A	853 (58.3)	976 (62.9)	1.00	
G/A	521 (35.6)	510 (32.8)	1.17 (1.00-1.36)	
G/G	88 (6.0)	67 (4.3)	1.51 (1.08-2.10)	
Dominant			1.21 (1.04-1.40)	0.011
Recessive			1.42 (1.03-1.97)	0.034
Additive			1.20 (1.06-1.35)	0.004
Combined genotypes (unfavorable genotypes carried) ^a^				
0	784 (55.5)	971 (62.8)	1.00	
1-3	553 (39.2)	514 (33.3)	1.34 (1.15-1.56)	<0.001
4-6	75 (5.3)	61 (4.0)	1.52 (1.07-2.16)	0.019

Note: The logistic regression analyses were adjusted for age and gender.

a The combined genotypes were trichotomized according to the unfavorable genotypes carried (rs3757328 AA, rs6940552 AA and rs9261204 GG were considered to be unfavorable genotypes). 0 indicates the presence of no unfavorable genotypes, 1–3 indicating the presence of 1–3 unfavorable genotypes, and 4-6 indicates the presence of 4-6 unfavorable genotypes.

Next, we estimated the cumulative effects of the three SNPs on HBV-related HCC susceptibility by adding the numbers of variant alleles. As summarized in Table 1, the HCC risk significantly and dose-dependently increased with the number of variant alleles (adjusted OR =1.34, 95% CI =1.15-1.56 for one to three variant alleles and adjusted OR =1.52, 95% CI = 1.07-2.16 for four to six variant alleles; *P* for trend <0.001), compared with the wild-type (WT) subjects who were homozygous for the three SNPs (Table 1).

**Figure 1:**
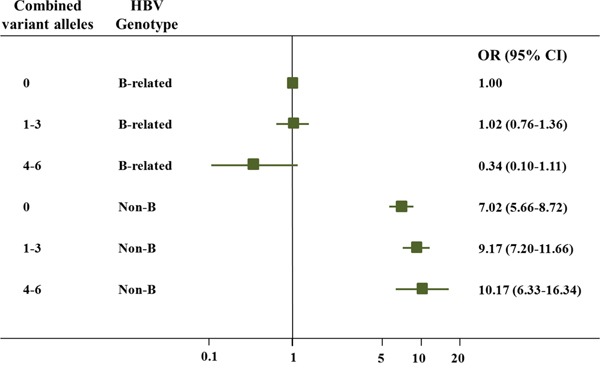
Crossover analysis of the effect of the three SNPs-HBV genotype interactions on HCC susceptibility The non-B groups with 0 alleles (i.e., rs3757328-G, rs6940552-G and rs9261204-A), 1–3 alleles, and 4–6 alleles were associated with significantly increased risks (adjusted OR = 7.02, 95% CI = 5.66-8.72, *P* < 0.001; adjusted OR = 9.17, 95% CI = 7.20-11.66, *P* < 0.001; and adjusted OR = 10.17, 95% CI = 6.33-16.34, *P* < 0.001, respectively) of chronic HBV infection, as compared with the most common combination (i.e., the B-related groups with “0” alleles).

The combined effects of the three SNPs on HCC susceptibility were also evaluated by stratifying on the basis of age, gender and HBV genotype (Table 2). Consequently, similar association strengths were found in most of the subgroups (*P* > 0.05 for heterogeneity test). Interestingly, a stronger combined effect of the three SNPs on HCC risk was observed among the non-B groups (adjusted OR = 1.26, 95% CI = 1.05-1.50) compared with the B-related groups (adjusted OR = 0.89, 95% CI = 0.69-1.15; *P* = 0.029 for the heterogeneity test). Further interactive analysis detected a significant multiplicative interaction between the variant alleles and the HBV genotypes on HCC susceptibility (*P* = 0.030; Table 3). Crossover analysis suggested that the non-B groups with 0 alleles (i.e., rs3757328-G, rs6940552-G and rs9261204-A), 1–3 alleles, and 4–6 alleles were associated with significantly increased risks (adjusted OR = 7.02, 95% CI = 5.66-8.72, *P* < 0.001; adjusted OR = 9.17, 95% CI = 7.20-11.66, *P* < 0.001; and adjusted OR = 10.17, 95% CI = 6.33-16.34, *P* < 0.001, respectively) of chronic HBV infection, as compared with the most common combination (i.e., the B-related groups with “0” alleles; Table 3, Figure 1).

**Table 2:** Stratified analyses of the combined variant alleles and HCC susceptibility

Variables	HCC susceptibility (0 / 1-3 / 4-6)			
	HCC patients	HBV persistent carriers	OR (95% CI)	P^*^
Age				
≤53	476/351/42	510/313/34	1.04 (0.86-1.25)	0.252
>53	308/202/33	461/201/27	1.23 (0.99-1.53)	
Gender				
Male	644/440/54	770/426/49	1.06 (0.91-1.25)	0.133
Female	140/113/21	201/88/12	1.39 (1.01-1.90)	
HBV Genotype				
B-related^†^	174/88/3	645/317/33	0.89 (0.69-1.15)	0.029
Non-B^¶^	594/446/72	317/183/27	1.26 (1.05-1.50)	

Note: The logistic regression analyses were adjusted for age, gender and HBV genotype (excluding the stratified factor in each stratum) in the additive genetic model.

* P-value for the heterogeneity test.

† B-related genotypes including B and BC.

¶ Non-B genotypes including C and D.

**Table 3:** Crossover analysis of the combined variant allele-HBV genotype interactions on HCC susceptibility

Variables	HBV genotype	HCC patients (n = 1507) N (%)	HBV persistent carriers (n = 1560) N (%)	OR (95% CI)	*P*
0	B-related^†^	174 (12.6)	645 (42.4)	1	
1-3	B-related^†^	88 (6.4)	317 (20.8)	1.02 (0.76-1.36)	0.916
4-6	B-related^†^	3 (0.2)	33 (2.2)	0.34 (0.10-1.11)	0.731
0	Non-B^¶^	594 (43.1)	317 (20.8)	7.02 (5.66-8.72)	<0.001
1-3	Non-B^¶^	446 (32.4)	183 (12.0)	9.17 (7.20-11.66)	<0.001
4-6	Non-B^¶^	72 (5.2)	27 (1.8)	10.17 (6.33-16.34)	<0.001
Interaction				^a^*P* = 0.030	

Note: The logistic regression analyses were adjusted for age and gender.

a *P* value for the multiplicative interaction.

† B-related genotypes including B and BC.

¶ Non-B genotypes including C and D.

## DISCUSSION

The development of HCC is a multistage process, and most HCCs arise from chronic hepatitis induced by HBV infection, particularly in China. The natural histories of hepatitis B virus infection are not uniform and are affected by several that include the HBV genotype [17]. Studies have found that genotype C is an independent risk factor for HCC development [18–20]. Viral genotype may be an independent and direct cause of hepatocarcinogenesis, which is the result of continuous necroinflammation [21]. In a previous study, we have also identified a significant association between the C genotype and the risk of HCC [16].

Previously, Cao et al have conducted a case-control study (1342 healthy controls, 327 HBV surface antigen (HBsAg) seroclearance subjects, and 1531 HCC patients) to assess the effects of *HLA-DQ* genetic polymorphisms, HBV genotypes, HBV mutations and their interactions on the risks of HCC. The results indicate that the rs9275319 variant genotype (GG) is significantly associated with an increased frequency of preS1 start codon mutations, which are HCC-risk mutations, in genotype C [22]. Recently, our group has detected significant interactions between *HLA-DQ/DR* rs9272105 and both of the HBV genotypes (*P* < 0.05 for each), as enabled by a large sample size and a high detection rate [16]. However, additional genes with potential interactions with HBV sequence variations need to be identified. Here, we detected significant interactions between the *ZRND1* eQTL SNP and the HBV genotypes and found that these interactions were associated with HCC risk in a large sample.

Previous study had evidenced that *ZNRD1*, in close proximity to the HLA-A locus, was cloned from the human MHC class I region [23]. Our previous study has varified the significant association between two *HLA-DQ/DR* SNPs found by former GWAS studies and HCC risk (OR = 1.31, 95%CI = 1.18-1.45 for rs9272105; OR = 0.66, 95%CI = 0.56-0.78 for rs9275319) [16]. As a clone from MHC class I region, *ZNRD1* eQTLs also appear the similar association with HCC risk. Furthermore, a series of studies have demonstrated that *ZNRD1* variation affects host susceptibility to virus acquisition [10, 24]. In our previous work, we have found that the down-regulation of *ZNRD1* reduced the expression level of HBV, which was a major risk factor for the progression of HBV infection; this finding was consistent with the protective roles of the variant alleles of the three SNPs on persistent HBV infection. Interestingly, the opposite findings have been reported in relation to tumorigenesis after persistent HBV infection [15]. Several studies have demonstrated that the up-regulation of *ZNRD1* may inhibit DNA damage and enhance DNA repair capacity, which has been identified as a novel negative modifier in carcinogenesis [11, 12]. As we all kown, genes in the HLA region on chromosome 6 (such as *HLA-DP* and *HLA-DQ*, which were expressed as surface antigens and determine the immune response to virus infection) were associated with HBV infection and progression [25]. Which is more evidence that *ZNRD1* and *HLA* play alike roles in cancer risk and virus infection.

Recently, a large number of lncRNAs have been identified by next-generation sequencing-based analyses in studies of complex diseases, and research on lncRNA genetic variants and biological function has been gaining increasing interest and emphasis in the literature [26, 27]. The dysregulation of multiple lncRNAs has been reported in HCCs, including *HEIH* and *HULC* [28, 29]. Moreover, our previous study has demonstrated that the genetic variants of *ZNRD1-AS1* may exhibit different effects on HBV infection and HCC development [15]. The SNPs in lncRNAs may regulate the expressions of localized lncRNAs and subsequently affect the expression or function of nearby genes. Our current study further revealed that *ZNRD1* eQTL SNPs may influence the HCC risks associated with the HBV genotypes.

The effect of *ZNRD1* eQTLs was particularly evident in non-B genotype HBV-infected patients. Compared with HBV genotype B, genotype C was more prone to cause chronic HBV infection/inflammation and HCC [6, 30]. Our previous study also showed the similar conclusions. Results in Table 4 showed that the three eQTLs was associated with an increased risk of HCC. In addition, its interaction with HBV non-B genotype was greater associated with HCC risk (Table 3). In brief, the three eQTLs in *ZNRD1-AS1* might predispose the HBV-infected patients to dysregulation of *ZNRD1* which affect viral replication and interaction with HBV genotype, thus contributing to HBV-induced hepatocarcinogenesis.

**Table 4:** Primers and probes used in TaqMan allelic discrimination

Polymorphism		Sequence (5′-3′)
rs3757328	Primer	F: TTTCTTGACTACTGCTAGCCTCACTT
		R: GGTGGTGGAACAGAGGAGCTT
	Probe	G: FAM-TCTGGCAGGAGTCGA-MGB
		A: HEX-TCTGGCAGAAGTC-MGB
rs6940552	Primer	F: TACATAGCTAGAAGCAGCATCTATAATCC
		R: GCTTTAGAGTGTCATTGGTATGAACAG
	Probe	G: FAM-TCACATAGGAATCACTG-MGB
		A: HEX-TCTCACATAAGAATCAC-MGB
rs9261204	Primer	F: TCCTTGCTCTGCTCTGCATTAT
		R: TGGTCTTTTAGTGGATGTTTTTGG
	Probe	G: FAM-ATTTATTGGGACAGTCGTA-MGB
		A: HEX-ATGGGTAAGATTTATTGAGAC-MGB

In summary, this large case-control study revealed that multiplicative interactions of *ZNRD1-AS1* SNPs with HBV genotypes significantly affect HCC risk and suggested that HBV genotypes and *ZNRD1-AS1* SNPs (rs3757328, rs6940552 and rs9261204) may serve as susceptibility biomarkers for the risk of HBV-related HCC. Additional well-designed prospective studies are warranted to validate and extend our findings.

## MATERIALS AND METHODS

### Patient samples

The ethical committees of all of the institutions involved in this study approved this case-control study. The subject enrollment has previously been described [16]. Briefly, the HCC cases were recruited from January 2006 to May 2014 at the First Affiliated Hospital of Nanjing Medical University (Nanjing, China), the Nantong Tumor Hospital (Nantong, China) and the Qidong Liver Cancer Institute (Qidong, China) in central and southern Jiangsu Province, China. The diagnosis of HCC was confirmed by a pathological examination and/or an alpha-fetoprotein elevation (> 400 ng/ml) combined with an imaging examination. HCV-related HCC patients were excluded. Ultimately, 1,507 HBV-related HCC patients agreed to participate in the study. Controls who were recognized as being persistent HBV carriers from 2009 to 2010 and were also from three cities in central and southern Jiangsu Province (48,417 subjects from Zhangjiagang, 43,563 subjects from Taixing and 57,192 subjects from Danyang) were recruited. Chronic HBV carriers were defined as subjects who were positive for both hepatitis B surface antigen (HBsAg) and antibodies against the hepatitis B core antigen (anti-HBc) but were negative for the HCV antibody (anti-HCV) at their second visit. Approximately 2,475 (5.11%), 3,413 (7.83%) and 5,587 (9.77%) chronic HBV carriers were identified from Zhangjiagang, Taixing and Danyang, respectively. In total, 1,560 cancer-free controls were randomly selected from these three cities and frequency-matched to the cases on the basis of age and sex. The demographic information of these selected controls, including age and gender, was collected via face-to-face interviews.

### Serological testing

HBsAg, anti-HB, anti-HBc, and anti-HCV antibodies were detected in the subjects' sera with enzyme-linked immunosorbent assays (Kehua Bio-Engineering Co., Ltd., Shanghai, China) according to the manufacturer's instructions, as described previously [31].

### Genotyping of the ZNRD1-AS1 SNPs

Genomic DNA was extracted from leukocyte pellets by traditional proteinase K digestion, phenol-chloroform extraction and ethanol precipitation. The SNPs rs3757328, rs6940552 and rs9261204 were genotyped using a TaqMan allelic discrimination assay on an ABI 7900 system (Applied Biosystems, La Jolla, CA). Information about the primers and probes is displayed in Table 4. The genotyping assays were performed blindly, and two blank (i.e., water) controls in each 384-well plate were used for quality control. More than 10% of the samples were randomly selected for repeat analyses, which yielded a 100% concordance rate. The genotyping success rates for these three SNPs were all above 98%.

### Identification of the HBV genotypes

We extracted HBV DNA from 200 μl HBV-positive serum samples with High Pure Viral Nucleic Acid kits (Roche Diagnostics GmbH, Mannheim, Germany) following the manufacturer's instructions. We used an improved nested multiplex PCR to identify the HBV genotypes. P1-S and P1-AS primers were used for the amplifications of the first round of the HBV genotypes A, B, C and D. Moreover, genotype primers were used for the amplifications of the A, B, C and D genotypes. The primers used are detailed in our previous study [16]. All genotyping assays were performed blindly with respect to the subjects' case or control status, and two blank (i.e., water) controls in each 96-well plate were used for quality control. More than 10% of the samples were randomly selected for confirmation by DNA sequencing. The success rates of the determinations of the HBV genotypes and subgenotypes for each area were all above 96%.

### Statistical analysis

The associations of the HBV genotypes with the SNP genotypes and HCC risk were estimated by computing the odds ratios (ORs) and their 95% confidence intervals (CIs) based on logistic regression analyses. The heterogeneities of the associations among the subgroups were assessed with the χ2-based Q test. All of the statistical analyses were performed with R software (version 2.13.0; the R Foundation for Statistical Computing), and *P* ≤ 0.05 from two-sided tests was considered to be statistically significant.
